# The effects of patient education programs on medication use among asthma and COPD patients: a propensity score matching with a difference-in-difference regression approach

**DOI:** 10.1186/s12913-015-0998-6

**Published:** 2015-08-17

**Authors:** Nazmi Sari, Meric Osman

**Affiliations:** Department of Economics, University of Saskatchewan, S7N5A5 Saskatoon, SK Canada; Saskatchewan Health Quality Council, Saskatoon, Canada

## Abstract

**Background:**

Adherence to medication is one of the critical determinants of successful management of chronic diseases including asthma and chronic obstructive pulmonary disease (COPD). Given that poor adherence with self-management medication is very common among asthma and COPD patients, interventions that improve the use of chronic disease management medications for this patient group have potential to generate positive health outcomes. In an effort to improve asthma and COPD care, the Lung Association of Saskatchewan has implemented an intervention by providing access to effective and high quality asthma and COPD education for both patients and health care professionals along with increasing access to spirometry.

By evaluating the impacts of this intervention, our purpose in this paper is to examine the effectiveness of spirometry use, and asthma and COPD education in primary care setting on medication use among asthma and COPD patients.

**Methods:**

At the time of the intervention, the Lung Association of Saskatchewan has not assigned a control group. Therefore we used a propensity score matching to create a control group using administrative health databases spanning 6 years prior to the intervention. Using Saskatchewan administrative health databases, the impacts of the intervention on use of asthma and COPD medications were estimated for one to four years after the intervention using a difference in difference regression approach.

**Results:**

The paper shows that overall medication use for the intervention group is higher than that of the control group. On average, intervention group uses more asthma and COPD drugs. Within the asthma and COPD drugs, this intervention creates a persistent effect over time in the form of higher utilization of chronic management drugs equivalent to $157 and $195 in a given year during four years after the intervention.

**Conclusions:**

The study suggests that effective patient education and increasing access to spirometry increases the utilization of chronic disease management drugs among asthma and COPD patients. This type of interventions with patient education focus has potential to save healthcare dollars by providing better disease management among this patient group.

**Electronic supplementary material:**

The online version of this article (doi:10.1186/s12913-015-0998-6) contains supplementary material, which is available to authorized users.

## Background

Adherence to medication is one of the critical determinants of successful management of most chronic diseases including asthma and chronic obstructive pulmonary disease (COPD). Studies, however, repeatedly emphasize that non-adherence to medication, especially non-compliance with self-management medication, is very common among asthma and COPD patients [1–3]. As an attempt to improve medication adherence, health outcome, and quality of life, various interventions that target asthma and COPD patients have been implemented [4–10]. Main goal of these interventions was to educate patients about all aspects of the disease, teach them coping skills to avoid triggers, and to train the patients in medications with specific emphasis on doses, frequency of administration, and possible side effects.

As shown in some studies recently reviewed in a Cochrane review [11], patient education interventions improve medication adherence, decrease emergency department (ED) visits, and hospital admissions. For instance, Bruzzese et al. show that an education based intervention implemented in a New York City school environment substantially reduces healthcare utilization among adolescence [5]. The result of this randomized control trial shows that the intervention participants experience a 28 % reduction in acute medical visits, a 49 % reduction in ED visits, and a 76 % reduction in hospitalizations compared to adolescence receiving usual care. While there are other studies reviewed in this Cochrane review also showing similar results [4, 12], Farber and Oliveria [10], and Bailey et al. [6] show that patient education programs targeting this patient group have no impact on health care utilization measured by ED visits and hospitalization. In addition to ED visits and rate of hospitalizations, Farber and Oliveria also examine the impact of the intervention on medication [10]. They find that the intervention group uses more (about one dispensation) asthma controller medication compared to the control group. There are also other studies indicating the impact of patient education interventions on medication use for management of asthma and COPD. In a school-based intervention for urban adolescents, Bruzzese et al. [5] report that the students in the intervention group relative to the control group reported significantly greater use of controller medication, and more confidence to manage their asthma. Subsequent studies also report that patient education program resulted in significantly better controller adherence and long acting beta agonist adherence, improved steroid inhaler compliance and higher cumulative controller medication dose [4, 7, 9, 10, 13].

As a body of literature indicates, inappropriate or insufficient medications among patients with chronic conditions can result in several negative consequences including treatment failure, unnecessary and more intensified therapy, costly diagnostic procedures and complications and hospitalization. Hence effective asthma and COPD management with consistent use of self-management medications not only decreases emergency care, and unscheduled doctor visits, but also improves quality of life, and reduces productivity losses and disability leaves (for a review of this literature see Nieuwlaat et al. [11]).

In an effort to improve asthma and COPD care and health outcomes, the Lung Association of Saskatchewan has implemented an intervention by providing access to effective, and high-quality asthma and COPD education for both patients and health care professionals along with increasing access to spirometry to increase early detection of these diseases. For this purpose, the Association has placed spirometry and the Certified Respiratory Educators (CREs) (certified healthcare professionals with specific focus on asthma and COPD care) in selected family physician offices in 2007. The objective of the intervention was to educate patients in managing their diseases.

By evaluating the impacts of the intervention conducted by the Lung Association, our purpose in this paper is to examine the effectiveness of spirometry use, and asthma and COPD education in primary care setting on medication use among asthma and COPD patients. Our results show that overall medication use for the intervention group is higher than that of the control group. On average, the intervention group uses more asthma and COPD drugs. Within the asthma and COPD drugs, the intervention with its patient education focus creates a persistent effect over time in the form of higher utilization of chronic management drugs (i.e. inhaled steroids (also known as inhaled corticosteroids), long acting beta agonists) while it has no effect for the utilization of other drugs.

## Methods

### Overview of the intervention

The main purpose of this intervention was to educate patients on how to manage their diseases. In January 2006, the Lung Association has started a pilot intervention by introducing CREs and spirometry to selected family physician clinics in a medium size city in Canada. In 2007, this intervention was fully implemented in family physician offices who expressed interest in accessing the education program for their patients with a diagnosis of asthma and/or COPD. Physicians who participated in the intervention recruited asthma and COPD patients to the intervention if the patients (1) have asthma and/or COPD and would highly benefit from the intervention, or (2) require validation of their diagnosis, or (3) have high risk of developing asthma and/or COPD. Appointments to see patients are made in the physicians’ clinics for initial and follow-up consultations.

Each patient visit started with spirometry administration and continued with one hour patient education conducted by the CREs. Each session included training and information regarding the pathophysiology of the disease, medications and inhaler techniques, environmental control for asthma, and coping skills. The intervention was patient oriented with a special focus on the needs of the patients. In order to meet the specific needs, it was tailored based on the needs of a corresponding patient. For instance, if the patient was a smoker, more time has been devoted to explain smoking cessation programs.

At the end of each session, patients have consulted with the physician for any change in medications and an action plan afterwards. Office/phone follow-ups within four weeks following the session were also scheduled for all patients. While phone follow-ups were around 15 min, office follow-ups were expected to last for about an hour. However, the duration of office follow-ups varied depending on the needs of the patient. Similarly, patients also had a chance to consult with the physician after the follow-up session. Certified educators were available to meet with the patients depending on the need and request by the patients after the first follow-up.

### Data source and intervention sample

Several data sets from the Saskatchewan health administrative databases were used for our analysis. An additional file shows the description of each database in more detail (see Appendix Table A.1. in Additional file 1). The outcome variables (i.e. prescription drug cost and use) were created using the Prescription Drug Plan Historical Claims (PDP) that includes details on prescription drug related information such as quantity of drug dispensed, date of dispensing, cost, type and name of the drugs with their drug identification numbers. Additional databases such as the Discharge Abstract Database (DAD) for patients’ hospitalizations, the Medical Services Billing Claims Data (MSB) for the physician visits, the Personal Health Registration System (PHRS) for patient demographics and location of residence, and Vital Statistics Registry for death information were utilized in this study.

All of the files can be linked with each other using the encrypted unique identifier (encrypted provincial health number) that are provided in each data set for all residents in the province. The Lung Association recorded the provincial health number for intervention participants and this was converted to the encrypted form using the same algorithm applied for encryption in all other databases. The common identifier was then used to obtain records for the intervention participants from the Saskatchewan administrative health databases.

The ethics approval for this study has been obtained from the University of Saskatchewan Biomedical Research Ethics Board under Bio # 10–180. The patients gave free and informed consent for linkage of data collected directly from them (i.e., that they were participants in the CRE program) and the administrative health data about their utilization.

There were 271 patients participated to the intervention in 2007. We restricted the intervention sample to the patients with health care utilization data available through the Saskatchewan administrative health databases. Among 271 patients in the initial sample, we dropped 28 patients due to missing de-identified health number, and 13 patients with Registered Indian status since there are no prescription drug data available for them. We also excluded 15 patients as they did not live in this health region during the pre-intervention period. We also excluded 9 patients who died during the study period. In addition, 13 patients who did not use any physician services or were younger than seven in the baseline year were also dropped. Finally, we further excluded 8 patients for which no match were found in our propensity score matching strategy. As a result, the final intervention sample used in our intervention effect estimations consists of 185 individuals^1^.

### Outcome variables

By following Kuwornu et al. [14], and HQC [15], we categorized the drugs under three groups (chronic management, acute, and other unclassified drugs). We grouped the drugs that are only used for chronic management (i.e. to control and improve symptoms) under *chronic management drug* group (i.e. inhaled steroids (also known as inhaled corticosteroids), long acting beta agonists (LABA), anticholinergic, and xanthine). The drugs that are only used for acute exacerbations were identified under *acute drugs (*i.e. short-acting beta-agonists (SABA), antibiotics). Following the study conducted by HQC [15], other asthma drugs that are not classified in chronic management or acute drugs by Kuwornu and his colleagues [14] were grouped under *unclassified asthma drugs*.

The cost of drugs is recorded in the databases in nominal terms. In order to compute the real prescription drug costs, we used 2002 as the base year, and adjusted the cost using the Saskatchewan Consumer Price Index for health and personal care commodity group.

### Other variables

A rich set of variables including key demographic variables was used in the propensity score matching method that is described in the next section. In order to capture patient heterogeneity due to their health status, we used comorbidity index based on the Charlson and Elixhauser indexes [16, 17]. The list of binary variables indicating the presence of corresponding comorbidities, and key demographic variables along with their descriptions are presented in the additional file (see Appendix Tables C.1-C.4 in Additional file 1).

### Statistical analysis

#### Propensity score matching

In order to estimate the effects of the intervention on the use of chronic disease management and acute drugs, one needs to compare the outcomes between intervention participants and comparable non-participants. Although we have the intervention group exposed to the intervention, no control group was assigned when the intervention was conducted by the Lung Association. To deal with this shortcoming, we constructed a control group using a propensity score (PS) matching strategy [18–20]. The purpose of the matching is to identify individuals that have similar characteristics to the intervention participants except for the intervention status.

Before estimating the propensity scores based on a rich set of covariates in a probit specification, we restricted the potential control group using key characteristics of the intervention group such as age and geographical location and data availability prior to the intervention. For this purpose, we restricted the potential control to the sample that includes only individuals who used any physician services during 2006, and who lived in the same health region with the intervention participants prior to the implementation of the intervention. We defined the period before the intervention from April 2001 to December 2006. The time period was chosen based on the availability of the data. We dropped individuals who died at any time since April 2001. We also constrained the sample to the individuals aged seven to ninety-six in 2006. Given that we have no prescription drug information available for people with Registered Indian status, we also dropped them from the analysis.

Following this stratification, we estimated a probit model to compute propensity scores. These scores were estimated for each remaining individual in the risk set using a probit model with a long list of baseline characteristics including the baseline outcome variables as well as health status measures such as comorbidity indicators for 19 conditions. These details are presented in the online appendix to this paper (see Appendix Table B.1. in Additional file 1). All statistical analysis outlined in this and next sub-sections were employed using Stata (version 12), and SAS (version 12) software programs.

There are various propensity score matching techniques used in the literature (for a review see Austin [21], Dehejia and Wahba [22]. The simplest one is the nearest neighbor matching in which an intervention unit is matched with a unit from the potential control that has the closest propensity score to the corresponding intervention unit. In the case of multiple matches, the tie is broken by a random draw. Even though this approach requires the propensity scores to be the closest between the intervention and matched control units, it has potential to create considerable bias. When we use it with replacement, the nearest neighbor matching can limit the number of matched controls which in turn may result in small sample bias. If it is used without replacement, then the quality of matched controls could be poor due to possible large differences in the propensity scores between the intervention unit, and the corresponding matched control [22].

In an effort to deal with these shortcomings, caliper (radius) matching is used as an alternative in the literature. This technique is simply an extension of the nearest neighbor matching that is developed to avoid the small sample bias, and the potential bias due to possible large differences in the propensity scores. This approach constructs the matched control units whose propensity scores are within a tolerated distance from the propensity score of the respective intervention unit. Formally the matched set *M*(*i*) for intervention unit *i* is defined as;

1 $$ M(i)=\left\{{p}_j\left|\kern1.25em \right|\left|{p}_i-{p}_j\right|\Big|<r\right\} $$

where subscripts *i* and *j* stand for the intervention and control units, respectively. The equation above denotes that all control units with estimated propensity scores, *p*_*j*_, fall within a radius *r* from the intervention unit *i*’s propensity score, *p*_*i*_, are matched to the intervention unit *i*.

In our study, we used a *with replacement* radius matching using a radius of 0.005. This is the smallest radius frequently used in the literature [23]. Given that there can be multiple control units within the specified radius, there are more than one match for each intervention unit. In order to deal with unlimited number of matches, we restricted the maximum number of matched controls for each intervention unit at 10. All potential controls with an estimated propensity score falling within a specified radius defined above were matched with the respective intervention unit. In the case of multiple matches due to identical propensity scores after nine successful matches, the tie was broken by a random draw among the last group of matched control units with identical propensity scores. We removed the rest of potential control units if their propensity scores were away from the tolerated distance from the propensity scores of any intervention units.

**Fig. 1:**
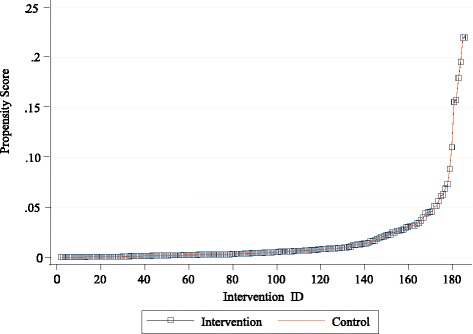
Distribution of propensity scores for intervention and control groups

Following the approach described above, we performed the matching and presented the distribution of propensity scores for both groups in Fig. 1. The vertical axis shows the probability of being in the intervention group for the intervention and control groups whereas the horizontal axis displays the observation identifier number for the intervention units. As there are multiple matches for individuals in the intervention group, the mean propensity scores of matched controls for the corresponding intervention unit, and the propensity score of each intervention unit are illustrated in the figure. The figure shows that the propensity scores for both groups are almost identical implying that both groups have the same propensity score distribution.

In order to assess the quality of matching based on observed characteristics as well as to understand the baseline values for outcome variables, we presented the baseline characteristics between the control and intervention groups. Table 1 shows the summary statistics for main variables with a mean difference test between the groups. As indicated in the table, the two groups have similar characteristics at baseline; the mean differences for each covariate between two groups are not statistically significant at 5 % significance level.

**Table 1:** Baseline comparisons for main outcome variables

	Intervention		Control		*p*-values for mean difference test
	Mean	SD	Mean	SD	
Physician services					
Cost (average cost in dollars/year)	559	550	549	670	0.83
Prescription drug cost (average cost in dollars/year)					
Asthma/COPD drugs	228	436	170	416	0.08
Asthma/COPD chronic management	149	354	110	335	0.13
Asthma/COPD acute drugs	62	104	50	115	0.18
Asthma drugs (unclassified)	17	123	10	65	0.23
Other drugs	841	1331	877	2789	0.86
Total drugs	1069	1490	1047	2840	0.92
Prescription drug dispensations (average count/year)					
Asthma/COPD drugs	5.2	7.44	4.1	7.09	0.06
Asthma/COPD chronic management	2.0	4.59	1.4	3.84	0.06
Asthma/COPD acute drugs	2.9	3.82	2.5	4.24	0.18
Asthma drugs (unclassified)	0.27	1.38	0.20	1.07	0.41
Other drugs	19.6	24.67	17.8	25.81	0.36
Total drugs	24.8	27.23	21.9	27.84	0.18
Hospital services (average count/year)					
Inpatient admission	0.10	0.36	0.08	0.35	0.63
Length of stay	0.59	2.44	0.39	2.46	0.29

Note: The mean shows the annual average for each variable. SD stands for standard deviations. The last column presents the *p-*values for the mean difference test between control and intervention groups. Costs for physician services and prescription drugs are measured in current dollars while drug dispensations and hospital services are measured in average annual counts of dispensations or encounters.

#### Regression framework to estimate the intervention effect

In order to estimate the effect of the intervention on use of chronic management asthma and COPD medications, we used a combined matching and a difference in difference (DID) regression approach rather than a matching estimator alone to estimate the intervention effect. An advantage of the combined matching-DID approach is that it relies on relatively weaker assumptions compared to the matching estimators alone. In the case of matching estimators, the estimated intervention effect relies on the independence assumption between the exposure to the intervention and potential health outcome upon controlling the relevant covariates [24]. One way to violate this assumption would be through unobservable factors affecting the selection to the intervention that in turn affects health outcome. The combined matching-DID approach removes any remaining, even after matching, time-invariant systematic differences between the intervention and control units. Although the combined approach eliminates any time invariant unobserved differences, it cannot provide a solution to any bias due to a change in health status for either group after the matching. One important health status indicator would be the new health conditions and comorbidities that are developed after the matching. With a combined matching-DID approach, we controlled for several comorbidities developed any time after the baseline as defined below:

2 $$ {y}_i=\alpha +\beta {I}_i+\gamma {D}_i+\delta {x}_i+{\varepsilon}_i $$

where subscript *i* stands for individuals who are either in intervention or control groups.

The dependent variable, *y*_*i*_ = *Y*_*i*1_ − *Y*_*i*0_, denotes the change in outcome variable *Y* before and after the intervention. In order to capture time variant differences between the intervention and control units, we used changes in the number of chest imaging services provided, physician consultation via telephone, non-clinical physician services (i.e. health teaching/counseling regarding patient’s treatment), and physician services received with provisional codes before and after the intervention. In addition, we used a matrix of indicator variables, *D*_*i*_, for several new conditions and comorbidities developed any time after the baseline.

In order to determine the intervention effects, we are specifically interested in estimating the coefficient of the indicator variable, *I*_*i*_, that identifies the intervention group. As a result, the model above was estimated separately for each year after the intervention to obtain one to four year intervention effects. We summarized main results for the intervention effect in the next section. The full results for each outcome variable are presented in the additional file (see Appendix Tables D.1-D.4, and G.1-G.4 presented in Additional file 1).

## Results

We examined the intervention effect on cost and dispensation of medications by type of the prescription drugs. For each outcome variable, we estimated model (2) for each year in order to identify the intervention effect in the corresponding year. Table 2 presents a summary result for the cost of prescription drugs within one to four years after the intervention while Table 3 presents the same information for drug dispensations.

**Table 2:** Summary results for the intervention effect on annual cost of prescription drugs

	2008	2009	2010	2011
Asthma/COPD drugs	192.6 **(0.00)**	192.2 **(0.00)**	204.9 **(0.00)**	156.6 **(0.00)**
Asthma/COPD chronic management	164.5 **(0.00)**	178.5 **(0.00)**	195.5 **(0.00)**	157.1 **(0.00)**
Asthma/COPD acute drugs	−6.45 (0.41)	−10.4 (0.18)	−6.09 (0.48)	−8.5 (0.40)
Other asthma drugs (unclassified)	34.6 (0.06)	24.1 (0.18)	15.5 (0.35)	8.03 (0.60)
Other drugs	−2.6 (0.97)	51.4 (0.53)	168.2 (0.15)	109.1 (0.38)
All drugs	190.0 **(0.04)**	243.6 **(0.02)**	373.2 **(0.01)**	265.7 (0.06)

Note: The numbers in parentheses show the *p-*values for the statistical significance of the estimated coefficient for the intervention effect. *P-*values for statistically significant results are shown in bold

**Table 3:** Summary results for the intervention effect on drug dispensations in counts

	2008	2009	2010	2011
Asthma/COPD drugs	2.12 **(0.00)**	1.95 **(0.00)**	2.01 **(0.00)**	1.45 **(0.03)**
Asthma/COPD chronic management	1.52 **(0.00)**	1.66 **(0.00)**	1.62 **(0.00)**	1.45 **(0.00)**
Asthma/COPD acute drugs	0.03 (0.91)	−0.12 (0.69)	0.11 (0.74)	−0.17 (0.60)
Other asthma drugs (unclassified)	0.58 **(0.02)**	0.41 (0.06)	0.27 (0.18)	0.17 (0.37)
Other drugs	−0.73 (0.54)	0.78 (0.55)	0.49 (0.71)	1.79 (0.24)
All drugs	1.39 (0.34)	2.73 (0.09)	2.50 (0.14)	3.23 (0.09)

Note: The numbers in parentheses show the *p-*values for the statistical significance of the estimated coefficient for the intervention effect. *P-*values for statistically significant results are shown in bold

Each column in Table 2 presents the intervention effect for the corresponding year. As shown in the table, overall drug cost for the intervention group is higher than that of the control group. On average, intervention group spends more on prescription drugs primarily due to asthma and COPD related drugs. However, there is no difference in the use of acute care asthma and COPD drugs. This change is primarily driven by the chronic management drugs.

The intervention creates a persistent effect over time in the form of higher utilization of chronic management drugs. In particular, Table 2 shows that the effect on the annual cost of chronic management drugs within one to four years following the intervention is between $157 and $195. As compared to the cost for chronic management drugs at the baseline, this effect is quite substantial. It implies that the intervention increases the cost by more than 100 % of the baseline cost for chronic management drugs of the intervention group.

We observe the same pattern over the same time period when we assess the dispensation of chronic management drugs in average number of annual prescription filled. As shown in Table 3, the intervention increases the dispensation of chronic management drugs. The effect varies from 1.4 to 1.6 prescription filled in a given year. This overall effect on chronic management drugs clearly indicates that the use of chronic management medications substantially increases with the intervention even four years after the intervention. For any other types of drugs, there is no evidence that the intervention has any impact on cost or number of prescription drug utilization.

## Discussions

Administrative health databases provide a unique opportunity to follow patients across multiple years and observe their drug dispensations. By comparing the intervention participants with a comparable control group we examined the impact of intervention on medication use, and other health care service utilization.

Our results suggest that intervention participants have consistently used more chronic management drugs during four consecutive years following the intervention. This finding related to the long-term sustainability of the intervention is an important contribution to the literature as previous studies reported that the initial increase in the use of chronic management drugs started to decrease after the active intervention period [4]. While we showed that intervention increases the use of chronic disease management drugs, it does not have any statistically significant effect on number of hospitalizations, length of hospital stays, and cost of physician visits. We presented a summary table showing these additional findings (see Appendix: Table 4). This implies that the intervention may generate saving since it is expected that patients who can manage their diseases better, are more likely to require fewer resources when hospitalized. Given that an average cost of an inpatient visit is $2,938 for asthma and $6,514 for COPD patients [25], even a small difference in required resources during inpatient treatment generates substantial net savings for the healthcare system.

As indicated earlier, a body of literature shows that better asthma and COPD management reduces unscheduled health care use [5, 13, 26]. Therefore, the patient education intervention studied in this paper may also generate an additional saving by reducing emergency care visits. The Canadian Institute for Health Information (CIHI) estimates that the average cost for a single asthma related emergency department visit is $205 while the average cost of a COPD related visit is $275 [25]. Our results suggest that the incremental cost of the intervention in the form of higher utilization of chronic management drugs is lower than the average cost of one emergency visit for this patient group. This implies that the intervention would create net saving if, on average, less than one emergency department visit per patient is avoided even if it has no impact on other health outcomes. However, emergency department data are not available for this study to directly measure whether emergency utilization decreases. Due to limitations in data availability for emergency care visits, and intensity of inpatient care for the study participants, we cannot examine these issues. These additional questions deserve further research.

In addition to the limitation mentioned above, there are other shortcomings of administrative health databases. For instance completeness and quality of administrative databases can be considered as another limitation. In our case, drugs administered in hospitals and over the counter purchases are not captured in the PDP database. As smoking cessation drugs are available over the counter, we are unable to fully capture the impact of these drugs on patient behavior regarding the use of other asthma and COPD drugs. These limitations need to be taken into consideration when interpreting the results documented in this paper.

## Conclusion

In this paper, we examined the effects of Lung Association’s intervention on asthma and COPD medications using propensity score matching and regression analysis. Our results show that the intervention improves medication use, especially for those that are used for management of asthma and COPD. The effect is significant and persistent during the study period covering four years after the intervention. However, there is no observed intervention effect on use of asthma and COPD acute care drugs, other asthma drugs, or utilization of other drugs which are not related to asthma and COPD. Our main result shows that the intervention increases the chronic management drug costs by about $157 to $195 in any year during the study period. The same pattern for dispensations of chronic management drugs were also observed in this study. Based on our findings, we conclude that an education program for asthma and COPD patients has potential to improve patients’ health outcome by increasing the use of chronic management medications.

## Appendix

Our additional results indicate that the intervention does not have any statistically significant effect on number of hospitalizations, length of hospital stays, and cost of physician visits. These findings are summarized in Table 4 below.

**Table 4:** Summary results for the intervention effect on physician and hospital services

	2008	2009	2010	2011
Average cost for physician services	2.02 (0.97)	10.68 (0.85)	−16.7 (0.76)	72.2 (0.25)
Hospital services				
Numbers of inpatient admissions	−0.06 (0.09)	−0.01 (0.77)	−0.01 (0.83)	0.08 (0.15)
Numbers of asthma/COPD inpatient admissions	−0.02 (0.19)	0.01 (0.69)	−0.01 (0.99)	0.01 (0.87)
Length of stay in number of days	−0.49 (0.14)	−0.44 (0.19)	−0.53 (0.31)	1.04 (0.28)

Note: The numbers in parentheses show the *p-*values for the statistical significance of the estimated coefficient for the intervention effect

## Additional file

Additional file 1:
**There is an online appendix uploaded in pdf format with a file name of** ***HSR_Online_appendix.*** It presents the full results from our empirical analysis including a brief description of databases used in this paper. (PDF 856 kb)

## Abbreviations

CIHI: Canadian Institute for Health Information
COPD: Chronic obstructive pulmonary disease
CREs: Certified Respiratory Educators
DAD: Discharge Abstract Database
ED: Emergency department
DID: Difference-in-difference
MSB: Medical services billing claims data
PDP: Prescription Drug Plan Historical Claims
PHRS: Personal Health Registration System
PS: Propensity score

## Footnotes

1 At the time of the intervention, the researchers of this paper were not part of the team conducting the intervention. Therefore they have not performed any power analysis to determine the sample size.
