# Supplementary material for: The effects of patient education programs on medication use among asthma and COPD patients: a propensity score matching with a difference-in-difference regression approach
Source: BMC Health Serv Res. 2015 Aug 17;15:332. doi: 10.1186/s12913-015-0998-6 (PMC4537780; doi:10.1186/s12913-015-0998-6)
Supplement: Additional file 1: — There is an online appendix uploaded in pdf format with a file name of HSR_Online_appendix. It presents the full results from our empirical analysis including a brief description of databases used in this paper. (PDF 856 kb) [file 12913_2015_998_MOESM1_ESM.pdf]

APPENDIX TABLES

Appendix Table A.1. Key variables in the Saskatchewan Health Administrative Databases

Dataset	*Key Variables from Dataset*
Person Health Registry System (PHRS)	Patient Health Card Number (encrypted)<br>Person year of birth<br>Gender<br>Registered Indian status<br>Status of health insurance coverage<br>Regional Health Authority where person resides
Hospital Discharge Abstract Database (DAD)	Patient Health Card Number (encrypted)<br>Date of admission<br>Date of discharge<br>Discharge diagnosis (ICD, all fields)<br>Mortality in hospital flag<br>Hospital name<br>Hospital category
Physician Services Claims File: Medical Services Branch (MSB)	Patient Health Card Number (encrypted)<br>Provider MSB number (encrypted)<br>Fee code approved<br>Diagnostic code (ICD or MSB) associated with service<br>Date of service<br>Number of services<br>Type of service or major group code<br>Location of service code
Prescription Drug Plan Historical Claims	Patient Health Card Number (encrypted)<br>Drug identification number (DIN)<br>Date of dispensing<br>Quantity of drug dispensed<br>Drug type (EDS, MSD)<br>Drug class (Major, minor)
Vital Statistics	Patient Health Card Number (encrypted)<br>Date of death

Appendix Table B.1. Propensity Score Estimations based on probit specification

Variable name	Coefficient	p-values	Variable name	Coefficient	p-values
Age	-0.01*	0.03	Slush Code Serv. – 2001	-0.13	0.59
Age square	0.01*	0.01	Slush Code Serv. – 2002	-0.03	0.54
Male	-0.27*	0.02	Slush Code Serv. – 2003	0.10	0.30
Age x Male	0.01	0.11	Slush Code Serv. – 2004	0.01	0.93
Urban	-0.19**	0.01	Slush Code Serv. – 2005	-0.03	0.83
No Diag. but Drug	0.22*	0.04	Slush Code Serv. – 2006	0.01	0.94
Pulmonary Func. Test – 2001	0.33	0.09	Other Slush Code Serv. – 2001	0.02	0.54
Pulmonary Func. Test – 2002	-0.29	0.26	Other Slush Code Serv. – 2002	-0.02	0.45
Pulmonary Func. Test – 2003	-0.18	0.42	Other Slush Code Serv. – 2003	0.01	0.48
Pulmonary Func. Test – 2004	-0.44	0.08	Other Slush Code Serv. – 2004	0.02	0.40
Pulmonary Func. Test – 2005	0.30*	0.04	Other Slush Code Serv. – 2005	-0.01	0.95
Pulmonary Func. Test – 2006	0.12	0.41	Other Slush Code Serv. – 2006	0.03*	0.05
Spirometry – 2001	0.05	0.50	Physician Costs – 2001	0.01	0.08
Spirometry – 2002	-0.03	0.66	Physician Costs – 2002	0.01	0.63
Spirometry – 2003	0.16*	0.01	Physician Costs – 2003	0.01	0.87
Spirometry – 2004	-0.12	0.24	Physician Costs – 2004	-0.01	0.07
Spirometry – 2005	0.04	0.54	Physician Costs – 2005	0.01**	0.01
Spirometry – 2006	0.16**	0.01	Physician Costs – 2006	-0.01	0.84
Imaging Serv. – 2001	-0.07	0.47	Myocardial Infarction	-0.53	0.07
Imaging Serv. – 2002	-0.11	0.17	Cong. Heart Failure	0.14	0.30
Imaging Serv. – 2003	-0.03	0.71	Peripheral Vascular	-0.35	0.14
Imaging Serv. – 2004	0.01	0.98	Cerebrovascular	-0.05	0.74
Imaging Serv. – 2005	0.03	0.51	Chronic Pulmonary	0.67***	0.00
Imaging Serv. – 2006	0.02	0.67	Connective Tissue	-0.31	0.10
Out province MD Vst. – 2001	-0.03	0.86	Peptic Ulcer	-0.07	0.56
Out province -MD Vst. – 2002	0.06	0.19	Mild Liver	0.06	0.82
Out province MD Vst. – 2003	-0.34	0.25	Diabetes w/o Complications	0.07	0.41
Out province MD Vst. – 2004	0.02	0.76	Paraplegia & Hemiplegia	-0.12	0.78
Out province MD Vst. – 2005	-0.04	0.72	Renal Disease	-0.40	0.15
Out province MD Vst. – 2006	-0.05	0.66	Cancer	-0.23	0.14
Non-Clinical MD Serv. – 2001	0.81***	0.00	Metastatic Carcinoma	-0.09	0.71
Non-Clinical MD Serv. – 2002	-0.03	0.92	Cardiac Arrhythmia	-0.04	0.84
Non-Clinical MD Serv. – 2003	-0.33	0.22	Hypertension (Uncomplicated)	0.01	0.91
Non-Clinical MD Serv. – 2004	0.35*	0.04	Hypothyroidism	0.05	0.54
Non-Clinical MD Serv. – 2005	-0.25	0.36	Obesity	0.46*	0.02
Non-Clinical MD Serv. – 2006	0.04	0.66	Weight Loss	0.05	0.92
Tel. Contact w. MD – 2001	0.36*	0.04	Depression/Psychoses	0.03	0.63
Tel. Contact w. MD – 2002	0.02	0.87	GP/FP Respiratory Serv. – 2001	0.02	0.56
Tel. Contact w. MD – 2003	-0.30	0.29	GP/FP . Respiratory Serv. – 2002	0.05*	0.03
Tel. Contact w. MD – 2004	-0.08	0.58	GP/FP Respiratory Serv. – 2003	0.01	0.77
Tel. Contact w. MD. – 2005	0.14	0.14	GP/FP Respiratory Serv. – 2004	0.01	0.74
Tel. Contact w. MD. – 2006	0.07	0.54			

Table B1. Continued

GP/FP Respiratory Serv. – 2005	0.01	(0.99	Hosp. Day Vst. – 2006	0.06	0.21
GP/FP Respiratory Serv. – 2006	0.02	0.38	Hosp. Inp. Resp. Vst – 2001	-0.12	0.78
Spec. Respiratory Serv. – 2001	-0.16	0.06	Hosp. Inp. Resp. Vst – 2002	0.14	0.54
Spec. Respiratory Serv. – 2002	-0.01	0.87	Hosp. Inp. Resp. Vst – 2003	-0.67	0.12
Spec. Respiratory Serv. – 2003	-0.04	0.51	Hosp. Inp. Resp. Vst – 2004	-0.08	0.76
Spec. Respiratory Serv. – 2004	0.01	0.20	Hosp. Inp. Resp. Vst – 2005	0.32	0.09
Spec. Respiratory Serv. – 2005	-0.034	0.45	Hosp. Inp. Resp. Vst – 2006	-0.12	0.51
Spec. Respiratory Serv. – 2006	-0.06	0.11	Hosp. Inp Other Vst – 2001	0.11	0.34
GP/FP Other Serv. – 2001	-0.01	0.34	Hosp. Inp Other Vst – 2002	-0.25	0.04
GP/FP Other Serv. – 2002	0.01	0.79	Hosp. Inp Other Vst – 2003	0.08	0.45
GP/FP Other Serv. – 2003	-0.01	0.53	Hosp. Inp Other Vst – 2004	-0.01	0.97
GP/FP Other Serv. – 2004	0.01	0.09	Hosp. Inp Other Vst – 2005	-0.05	0.71
GP/FP Other Serv. – 2005	-0.01	0.21	Hosp. Inp Other Vst – 2006	-0.15	0.20
GP/FP Other Serv. – 2006	0.01	0.53	Asthma Drugs (Unc.) – 2001	-0.03	0.81
Spec. Other Serv. – 2001	-0.02	0.15	Asthma Drugs (Unc.) – 2002	-0.05	0.52
Spec. Other Serv. – 2002	0.01	0.51	Asthma Drugs (Unc.) – 2003	0.06	0.47
Spec. Other Serv. – 2003	0.01	0.94	Asthma Drugs (Unc.) – 2004	-0.03	0.76
Spec. Other Serv. – 2004	0.02*	0.03	Asthma Drugs (Unc.) – 2005	-0.01	0.92
Spec. Other Serv. – 2005	-0.04**	0.00	Asthma Drugs (Unc.) – 2006	0.01	0.99
Spec. Other Serv. – 2006	0.01	0.90	Saba & Comb. Drugs – 2001	-0.03	0.58
LOS – 2001	-0.01	0.30	Saba & Comb. Drugs – 2002	-0.01	0.93
LOS – 2002	0.01	0.89	Saba & Comb. Drugs – 2003	-0.04	0.32
LOS – 2003	-0.01	0.48	Saba & Comb. Drugs – 2004	0.02	0.50
LOS – 2004	0.01	0.98	Saba & Comb. Drugs – 2005	-0.03	0.41
LOS – 2005	-0.03	0.28	Saba & Comb. Drugs – 2006	0.09	0.00
LOS – 2006	-0.01	0.90	Antibiotics – 2001	-0.05	0.31
Hosp. Day Vst. – 2001	-0.04	0.59	Antibiotics – 2002	-0.03	0.48
Hosp. Day Vst. – 2002	-0.04	0.50	Antibiotics – 2003	0.01	0.81
Hosp. Day Vst. – 2003	0.02	0.73	Antibiotics – 2004	-0.04	0.40
Hosp. Day Vst. – 2004	-0.03	0.64	Antibiotics – 2005	0.01	0.77
Hosp. Day Vst. – 2005	0.08	0.15	Antibiotics – 2006	0.01	0.78
Corticosteroids – 2001	0.04	0.82	Antibiotic Costs – 2001	0.01	0.01
Corticosteroids – 2002	0.04	0.83	Antibiotic Costs – 2002	-0.01	0.20
Corticosteroids – 2003	-0.03	0.86	Antibiotic Costs – 2003	-0.01	0.84
Corticosteroids – 2004	0.02	0.82	Antibiotic Costs – 2004	0.01	0.51
Corticosteroids – 2005	0.06	0.58	Antibiotic Costs – 2005	-0.01	0.94
Corticosteroids – 2006	-0.07	0.41	Antibiotic Costs – 2006	0.01	0.99
Chronic Man. Drugs – 2001	-0.01	0.84	Corticosteroid Costs – 2001	-0.01	0.65
Chronic Man. Drugs – 2002	0.01	0.81	Corticosteroid Costs – 2002	-0.01	0.60
Chronic Man. Drugs – 2003	0.04	0.42	Corticosteroid Costs – 2003	-0.01	0.97
Chronic Man. Drugs – 2004	-0.06	0.14	Corticosteroid Costs – 2004	0.01	0.80
Chronic Man. Drugs – 2005	0.01	0.80	Corticosteroid Costs – 2005	-0.01	0.68
Chronic Man. Drugs – 2006	-0.06	0.14	Corticosteroid Costs – 2006	0.01	0.20
Other Drugs – 2001	-0.01	0.08	Chronic Man. Drug Costs – 2001	0.01	0.42
Other Drugs – 2002	0.01	0.39	Chronic Man. Drug Costs – 2002	0.01	0.84
Other Drugs – 2003	0.01	0.36	Chronic Man. Drug Costs – 2003	-0.01	0.17
Other Drugs – 2004	-0.01	0.16	Chronic Man. Drug Costs – 2004	0.01	0.16

Table B1. Continued

Other Drugs – 2005	0.01	0.19	Chronic Man. Drug Costs – 2005	0.01	0.56
Other Drugs – 2006	0.01	0.93	Chronic Man. Drug Costs – 2006	-0.01	0.05
Asthma Drug (Unc.) Costs – 2001	-0.01	0.32	Other Drug Costs – 2001	-0.01	0.40
Asthma Drug (Unc.) Costs – 2002	0.01	0.11	Other Drug Costs – 2002	-0.01	0.99
Asthma Drug (Unc.) Costs – 2003	0.01	0.55	Other Drug Costs – 2003	-0.01	0.83
Asthma Drug (Unc.) Costs – 2004	-0.01	0.35	Other Drug Costs – 2004	0.01	0.11
Asthma Drug (Unc.) Costs – 2005	0.01	0.71	Other Drug Costs – 2005	-0.01	0.20
Asthma Drug (Unc.) Costs – 2006	-0.01	0.77	Other Drug Costs – 2006	0.01	0.80
Saba & Comb. Drug Costs – 2001	0.01	0.83	Constant	-3.22***	0.00
Saba & Comb. Drug Costs – 2002	-0.01	0.69			
Saba & Comb. Drug Costs – 2003	0.01	0.73			
Saba & Comb. Drug Costs – 2004	-0.01	0.45			
Saba & Comb. Drug Costs – 2005	0.01	0.38			
Saba & Comb. Drug Costs – 2006	-0.01	0.21			

Note: Number of observation is 167,513 individuals. * p-value≤0.05, ** p-value≤0.01, *** p-value≤0.001

Appendix Table C.1. Baseline Cost and Dispensations of Prescription Drugs

	Treatment (N=185)	Control (N =1,726)	
**Variable**	**Mean (SD)**	**Mean (SD)**	**Mean Difference Test (p-value)**
Chronic Man. Drugs - Total number of dispensed Asthma/COPD related chronic management drugs			
2001	1.16 (2.81)	0.83 (2.31)	0.07
2002	1.53 (3.88)	1.06 (2.94)	0.04
2003	1.38 (3.41)	1.00 (2.93)	0.10
2004	1.49 (3.41)	1.07 (3.05)	0.08
2005	1.87 (4.43)	1.39 (3.68)	0.10
2006	2.02 (4.59)	1.44 (3.84)	0.06
Acute Drugs - Total number of dispensed drugs for acute exacerbations			
2001	1.34 (2.28)	1.18 (2.52)	0.42
2002	1.65 (2.92)	1.49 (3.09)	0.50
2003	1.74 (2.95)	1.65 (3.30)	0.74
2004	2.05 (3.51)	1.85 (3.64)	0.47
2005	2.50 (4.07)	2.17 (3.89)	0.28
2006	2.91 (3.82)	2.47 (4.24)	0.18
Asthma Drugs (Unc.) - Total number of dispensed asthma related (unclassified) drugs			
2001	0.11 (0.74)	0.08 (0.59)	0.53
2002	0.27 (1.70)	0.16 (0.99)	0.17
2003	0.32 (1.72)	0.20 (1.05)	0.13
2004	0.21 (1.36)	0.13 (0.87)	0.31
2005	0.24 (1.32)	0.17 (1.05)	0.36
2006	0.27 (1.38)	0.20 (1.07)	0.41
Asthma/COPD Drugs - Total number of all Asthma/COPD related dispensed drugs			
2001	2.61 (4.81)	2.09 (4.31)	0.13
2002	3.46 (6.78)	2.70 (5.49)	0.08
2003	3.45 (6.09)	2.85 (5.54)	0.16
2004	3.75 (6.38)	3.06 (5.95)	0.14
2005	4.62 (7.56)	3.74 (6.84)	0.10
2006	5.20 (7.44)	4.12 (7.09)	0.06
Other Drugs - Total number of other (non-Asthma/COPD related) dispensed drugs			
2001	8.15 (11.99)	7.20 (11.60)	0.29
2002	13.11 (17.45)	12.25 (19.44)	0.56
2003	14.90 (21.60)	13.74 (22.88)	0.51
2004	16.10 (21.94)	14.82 (22.64)	0.46
2005	17.75 (22.62)	16.82 (24.74)	0.63
2006	19.60 (24.67)	17.80 (25.81)	0.36
Total Drugs - Total number of all dispensed drugs (Asthma/COPD Drugs + Other Drugs)			
2001	10.76 (18.83)	9.29 (12.81)	0.14
2002	16.57 (19.84)	14.95 (20.99)	0.31
2003	18.35 (23.03)	16.59 (24.27)	0.35
2004	19.85 (23.69)	17.88 (24.19)	0.29
2005	22.36 (25.24)	20.56 (26.79)	0.38
2006	24.80 (27.23)	21.92 (27.84)	0.18
Costs			
Chronic Man. Drug Costs - Total cost of dispensed Asthma/COPD related chronic management drugs			
2001	69.97 (172.41)	48.72 (145.28)	0.06
2002	94.71 (246.01)	63.71 (192.02)	0.04
2003	90.66 (237.44)	63.05 (197.62)	0.08
2004	109.16 (280.9)	76.30 (239.48)	0.08
2005	136.53 (352.09)	103.63 (310.74)	0.18
2006	148.90 (354.32)	109.94 (334.46)	0.13

Acute Drug Costs - Total cost of dispensed drugs for acute exacerbations			
2001	24.68 (57.29)	19.78 (57.34)	0.27
2002	27.73 (70.56)	23.43 (60.98)	0.37
2003	32.32 (75.47)	27.98 (78.35)	0.46
2004	37.86 (83.19)	31.60 (83.26)	0.33
2005	51.72 (121.43)	41.31 (99.91)	0.19
2006	62.14 (103.54)	50.35 (114.47)	0.18
Asthma Drug (Unc.) Costs - Total cost of dispensed asthma related (unclassified) drugs			
2001	4.75 (32.14)	3.48 (32.03)	0.61
2002	15.77 (110.66)	7.31 (54.99)	0.08
2003	17.65 (110.23)	8.53 (57.61)	0.07
2004	11.64 (82.66)	5.60 (41.95)	0.10
2005	12.77 (85.07)	7.55 (55.97)	0.26
2006	16.80 (122.48)	10.11 (65.15)	0.23
Asthma/COPD Drug Costs - Total cost of all Asthma/COPD related dispensed drugs (Chronic. Man. +acute + Asthma (Unc.) Drug Costs)			
2001	99.39 (215.14)	71.97 (185.17)	0.06
2002	138.22 (339.71)	94.15 (245.00)	0.03
2003	140.64 (331.45)	99.46 (258.72)	0.05
2004	158.66 (350.38)	113.50 (296.37)	0.06
2005	201.02 (426.19)	152.49 (386.88)	0.11
2006	227.84 (436.28)	170.42 (415.88)	0.08
Other Drug Costs - Total cost of other (non-Asthma/COPD related) dispensed drugs			
2001	266.19 (480.82)	238.96 (544.56)	0.51
2002	470.05 (852.21)	463.11 (1,142.67)	0.93
2003	621.05 (1,197.91)	643.32 (2,061.79)	0.89
2004	705.83 (1,439.59)	757.47 (2,696.86)	0.80
2005	719.55 (1,119.83)	805.2 (2,766.35)	0.68
2006	841.28 (1,331.30)	876.84 (2,789.01)	0.86
Total Drug Costs - Total cost of all dispensed drugs (Asthma/COPD + Other drugs)			
2001	365.58 (552.63)	310.94 (588.40)	0.23
2002	608.26 (971.12)	557.25 (1,189.38)	0.57
2003	761.69 (1,288.85)	742.78 (2,093.36)	0.90
2004	864.49 (1,505.89)	870.97 (2,728.22)	0.97
2005	920.57 (1,264.14)	957.71 (2,816.47)	0.86
2006	1069.12 (1,490.32)	1047.27 (2,840.15)	0.92

Appendix Table C.2. Baseline Hospital and Physician Service utilizations

Variable	Treatment (N=185) Mean (SD)	Control (N =1,726) Mean (SD)	Mean Difference Test (p-value)
Physician Services			
GP/FP Respiratory Serv. - Total number of respiratory related services provided by a family physician			
2001	0.64 (1.57)	0.57 (1.54)	0.52
2002	0.90 (1.77)	0.80 (1.83)	0.46
2003	0.85 (1.73)	0.79 (1.87)	0.69
2004	0.88 (1.74)	0.76 (1.83)	0.38
2005	1.03 (1.91)	0.85 (1.81)	0.19
2006	1.00 (1.86)	0.86 (1.75)	0.29
Spec. Respiratory Serv. - Total number of respiratory related services provided by a specialist			
2001	0.06 (0.54)	0.06 (0.44)	0.48
2002	0.13 (1.13)	0.12 (0.76)	0.84
2003	0.11 (0.78)	0.12 (0.82)	0.91
2004	0.17 (0.83)	0.14 (0.82)	0.63
2005	0.24 (1.22)	0.16 (0.89)	0.28
2006	0.26 (1.02)	0.18 (1.00)	0.32
GP/FP Other Serv. - Total number of other (non-respiratory) related services provided by a family physician			
2001	5.50 (5.66)	5.30 (6.39)	0.67
2002	7.94 (7.88)	7.97 (9.87)	0.97
2003	8.36 (6.98)	8.18 (10.29)	0.81
2004	8.93 (8.44)	8.76 (10.90)	0.84
2005	9.15 (7.38)	8.94 (10.62)	0.80
2006	9.38 (7.67)	9.22 (10.99)	0.84
Spec. Other Serv. - Total number of other (non-respiratory) related services provided by a specialist			
2001	2.94 (6.04)	2.82 (5.57)	0.80
2002	4.05 (6.38)	4.33 (8.79)	0.67
2003	4.31 (6.78)	4.32 (7.70)	0.99
2004	4.10 (5.63)	3.98 (6.88)	0.82
2005	3.86 (5.40)	3.66 (6.05)	0.65
2006	4.81 (6.57)	4.72 (7.91)	0.89
Costs			
Physician Costs - Total cost of all family physician and specialist services			
2001	288.00 (482.12)	278.70 (446.3)	0.79
2002	394.89 (518.6)	408.94 (593.2)	0.76
2003	433.65 (576.6)	415.11 (548.1)	0.66
2004	439.78 (421.4)	420.97 (482.3)	0.59
2005	508.68 (516.7)	479.54 (613.1)	0.53
2006	559.36 (550.2)	548.65 (670.1)	0.83
Hospital Visits			
Hosp. Day Vst. - Total number of day (outpatient) hospital visits			
2001	0.11 (0.49)	0.10 (0.34)	0.54
2002	0.16 (0.51)	0.15 (0.49)	0.78
2003	0.19 (0.44)	0.18 (0.54)	0.78
2004	0.16 (0.60)	0.16 (0.60)	0.89
2005	0.20 (0.56)	0.20 (0.67)	0.97
2006	0.24 (0.61)	0.24 (0.73)	0.95
Hosp. Inp. Resp. Vst. – Total number of inpatient hospital visits where a respiratory diagnosis is coded anywhere on patient's abstract			
2001	0.01 (0.07)	0.01 (0.07)	0.88
2002	0.03 (0.19)	0.02 (0.16)	0.80
2003	0.00 (0.00)	0.01 (0.08)	0.34
2004	0.02 (0.13)	0.01 (0.14)	0.87
2005	0.05 (0.28)	0.02 (0.17)	0.06
2006	0.03 (0.18)	0.01 (0.13)	0.06

Hosp. Inp. Other. Vst. – Total number of inpatient hospital visits where a respiratory diagnosis is not coded anywhere on patient's abstract			
2001	0.09 (0.45)	0.09 (0.36)	0.92
2002	0.06 (0.25)	0.06 (0.27)	0.87
2003	0.11 (0.48)	0.10 (0.39)	0.61
2004	0.08 (0.31)	0.07 (0.32)	0.75
2005	0.06 (0.26)	0.06 (0.27)	0.86
2006	0.06 (0.31)	0.07 (0.32)	0.79
LOS - Total length of hospital stays (in days)			
2001	0.45 (3.06)	0.38 (2.26)	0.69
2002	0.54 (3.29)	0.63 (5.74)	0.82
2003	0.49 (2.10)	0.48 (2.97)	0.96
2004	0.52 (2.99)	0.40 (3.08)	0.63
2005	0.45 (1.85)	0.30 (1.70)	0.25
2006	0.59 (2.44)	0.39 (2.46)	0.29

Note: S: cells are suppressed due to privacy.

Appendix Table C.3. Baseline Demographic and Comorbidity Characteristics

Variable	Treatment (N=185) Mean (SD)	Control (N =1,726) Mean (SD)	Mean Difference Test (p-value)
Demographic Characteristics			
Age - Age in 2006	47.43 (21.9)	46.45 (22.0)	0.57
Male - 1 if male, 0 if female	0.36 (0.04)	0.36 (0.01)	0.97
Urban - whether the person lived in an urban area (=1) - majority of the time - during the pre-treatment period			
2001- 2006	0.75 (0.03)	0.77 (0.01)	0.51
No Diag. but Drug - whether the person who did not diagnosed with asthma/COPD (pre-treatment) however, used at least one asthma/COPD related drug			
2001 - 2006	0.20 (0.03)	0.20 (0.01)	0.94
Comorbidities (22 Conditions)			
Myocardial Infarction - 1 if diagnosed with "myocardial infarction" during the pre-treatment period, 0 otherwise			
2001 -2006	<3% (0.10)[S]	0.01 (0.08)	0.41
Cong. Heart Failure - 1 if diagnosed with "congestive heart failure" during the pre-treatment period, 0 otherwise			
2001 - 2006	0.07 (0.25)	0.06 (0.22)	0.27
Peripheral Vascular - 1 if diagnosed with "peripheral vascular disease" during the pre-treatment period, 0 otherwise			
2001 -2006	<3% (0.12)[S]	0.01 (0.08)	0.10
Cerebrovascular - 1 if diagnosed with "cerebrovascular disease" during the pre-treatment period, 0 otherwise			
2001 -2006	0.04 (0.19)	0.03 (0.18)	0.83
Dementia - 1 if diagnosed with "dementia" during the pre-treatment period, 0 otherwise			
2001 -2006	0 (0)	0.01 (0.06)	0.39
Chronic Pulmonary - 1 if diagnosed with "chronic obstructive pulmonary disease" during the pre-treatment period, 0 otherwise			
2001 -2006	0.76 (0.43)	0.75 (0.43)	0.94
Connective Tissue - 1 if diagnosed with "connective tissue/rheumatic disease" during the pre-treatment period, 0 otherwise			
2001 -2006	<3% (0.14)[S]	0.02 (0.14)	0.99
Peptic Ulcer - 1 if diagnosed with "peptic ulcer disease" during the pre-treatment period, 0 otherwise			
2001 -2006	0.06 (0.23)	0.06 (0.23)	0.90
Mild Liver - 1 if diagnosed with "mild liver disease" during the pre-treatment period, 0 otherwise			
2001 -2006	<3% (0.12)[S]	0.01 (0.11)	0.75
Diabetes w/o Complications - 1 if diagnosed with "diabetes without complications" during the pre-treatment period, 0 otherwise			
2001 -2006	0.15 (0.35)	0.14 (0.35)	0.63
Diabetes w. Complications - 1 if diagnosed with "diabetes with complications" during the pre-treatment period, 0 otherwise			
2001 -2006	0 (0)	0.01 (0.09)	0.22
Paraplegia & Hemiplegia - 1 if diagnosed with "paraplegia and hemiplegia" during the pre-treatment period, 0 otherwise			
2001 -2006	<3% (0.07)[S]	<0.4% (0.05)[S]	0.56
Renal Disease - 1 if diagnosed with "renal disease" during the pre-treatment period, 0 otherwise			
2001 -2006	<3% (0.10)[S]	0.02 (0.12)	0.61
Cancer - 1 if diagnosed with "cancer" during the pre-treatment period, 0 otherwise			
2001 -2006	0.04 (0.19)	0.03 (0.18)	0.66
Moderate/Severe Liver - 1 if diagnosed with "moderate or severe liver disease" during the pre-treatment period, 0 otherwise			
2001 -2006	0 (0)	<0.4% (0.02)[S]	0.74
Metastatic Carcinoma. - 1 if diagnosed with "metastatic carcinoma" during the pre-treatment period, 0 otherwise			
2001 -2006	<3% (0.12)[S]	0.01 (0.12)	0.85
Cardiac Arrhythmia. - 1 if diagnosed with "cardiac arrhythmia " during the pre-treatment period, 0 otherwise			
2001 -2006	<3% (0.16)[S]	0.02 (0.14)	0.86
Hypertension (Uncomplicated) - 1 if diagnosed with "hypertension (uncomplicated)" during the pre-treatment period, 0 otherwise			
2001 -2006	0.39 (0.49)	0.36 (0.48)	0.39
Hypothyroidism - 1 if diagnosed with "hypothyroidism" during the pre-treatment period, 0 otherwise			
2001 -2006	0.10 (0.31)	0.10 (0.30)	0.96
Obesity - 1 if diagnosed with "obesity" during the pre-treatment period, 0 otherwise			
2001 -2006	<3% (0.16)[S]	0.03 (0.16)	0.94
Weight Loss - 1 if diagnosed with "weight loss" during the pre -treatment period, 0 otherwise			
2001 -2006	<3% (0.07)[S]	<0.4% (0.05)[S]	0.43
Depression & Psychoses - 1 if diagnosed with "depression or psychoses" during the pre-treatment period, 0 otherwise			
2001 -2006	0.26 (0.44)	0.25 (0.43)	0.92

Note: S: cells are suppressed due to privacy.

Appendix Table C.4. Other Baseline Characteristics

Variable	Treatment (N=185) Mean (SD)	Control (N =1,726) Mean (SD)	Mean Difference Test (p-value)
Other Patient Characteristics			
Pulmonary Func. Test - Total number of full pulmonary function tests taken			
2001	0.03 (0.18)	0.02 (0.15)	0.31
2002	0.02 (0.13)	0.01 (0.11)	0.69
2003	0.02 (0.15)	0.02 (0.14)	0.82
2004	0.02 (0.18)	0.01 (0.12)	0.42
2005	0.06 (0.26)	0.05 (0.23)	0.42
2006	0.05 (0.23)	0.03 (0.17)	0.13
Spirometry - Total number of spirometry taken			
2001	0.08 (0.49)	0.07 (0.43)	0.77
2002	0.08 (0.53)	0.08 (0.48)	0.89
2003	0.11 (0.57)	0.13 (0.64)	0.62
2004	0.09 (0.47)	0.06 (0.37)	0.35
2005	0.12 (0.75)	0.10 (0.51)	0.59
2006	0.20 (0.72)	0.20 (0.74)	0.86
Imaging Serv. - Total number of diagnostic radiology (x-rays) services to chest (with and without fluoroscopy, and bronchogram (unilateral))			
2001	0.09 (0.31)	0.09 (0.32)	0.82
2002	0.11 (0.33)	0.11 (0.36)	0.95
2003	0.14 (0.40)	0.14 (0.40)	0.91
2004	0.16 (0.38)	0.15 (0.46)	0.81
2005	0.23 (0.59)	0.22 (0.62)	0.73
2006	0.23 (0.58)	0.25 (0.69)	0.80
Out Province MD Vst. - Total number of out of province family physician and/or specialist visits			
2001	0.01 (0.07)	0.01 (0.18)	0.91
2002	0.12 (1.41)	0.18 (2.79)	0.78
2003	0.01 (0.07)	0.01 (0.10)	0.86
2004	0.13 (1.36)	0.22 (4.05)	0.75
2005	0.02 (0.29)	0.02 (0.25)	0.99
2006	0.03 (0.27)	0.03 (0.45)	0.92
Non-Clinical MD Serv. - Total number of non-clinical physician services (i.e. health teaching/counseling regarding treatment)			
2001	0.02 (0.15)	0.02 (0.16)	0.77
2002	0.01 (0.10)	0.02 (0.19)	0.74
2003	0.01 (0.07)	0.01 (0.07)	0.88
2004	0.03 (0.22)	0.02 (0.20)	0.61
2005	0.01 (0.10)	0.01 (0.16)	0.86
2006	0.05 (0.37)	0.02 (0.20)	0.04*
Tel. Contact w. MD - Total number of telephone contacts with the physician (i.e. advice/client counseling)			
2001	0.02 (0.16)	0.02 (0.25)	0.80
2002	0.03 (0.21)	0.02 (0.24)	0.61
2003	0.01 (0.07)	0.01 (0.10)	0.78
2004	0.03 (0.22)	0.01 (0.18)	0.35
2005	0.04 (0.45)	0.02 (0.30)	0.39
2006	0.04 (0.45)	0.03 (0.32)	0.63
Slush Code Serv.- Total number of services received with a slush (multipurpose) codes			
2001	0.01 (0.07)	0.01 (0.15)	0.76
2002	0.12 (1.55)	0.14 (2.10)	0.90
2003	0.02 (0.22)	0.03 (0.38)	0.70
2004	0.12 (1.40)	0.20 (1.40)	0.77
2005	0.02 (0.22)	0.02 (0.21)	0.97
2006	0.03 (0.23)	0.04 (0.49)	0.90
Other Slush Code Serv. - Total number of services received with other slush (multipurpose) codes			
2001	0.28 (1.44)	0.19 (1.29)	0.36

2002	0.39 (1.57)	0.24 (1.92)	0.30
2003	0.57 (1.87)	0.42 (2.08)	0.35
2004	0.65 (1.77)	0.53 (2.78)	0.68
2005	0.59 (1.44)	0.49 (3.04)	0.68
2006	0.81 (2.21)	0.62 (3.05)	0.42

Appendix Table D.1 Regression results for cost outcomes, 2006 vs. 2008

	Chronic Man. Drugs	Acute Drugs	Asthma Drugs (Unc.)	Asthma & COPD Drugs	Other Drugs	Total Drugs
Intervention effect	164.53***	-6.45	34.56	192.64***	-2.61	190.03*
	(0.00)	(0.41)	(0.06)	(0.00)	(0.97)	(0.04)
Tel. Contact w. MD	123.98	4.42	-2.26	126.14	133.87	260.01*
	(0.10)	(0.38)	(0.70)	(0.11)	(0.06)	(0.05)
Non-Clinical MD Serv.	-24.71	-5.73	-16.59*	-47.03	48.57	1.55
	(0.47)	(0.26)	(0.04)	(0.22)	(0.50)	(0.99)
Imaging Serv.	78.85**	6.45	9.77	95.06***	-14.49	80.57
	(0.01)	(0.19)	(0.26)	(0.00)	(0.63)	(0.06)
Other Slush Code Serv.	-1.18	-1.02	-0.77	-2.96	2.02	-0.94
	(0.71)	(0.08)	(0.32)	(0.39)	(0.79)	(0.91)
Myocardial Infarction	175.67	46.38	-41.93	180.12	350.69	530.81
	(0.18)	(0.26)	(0.10)	(0.27)	(0.10)	(0.09)
Cong. Heart Failure	47.10	2.01	0.69	49.79	152.33	202.13
	(0.45)	(0.94)	(0.97)	(0.53)	(0.24)	(0.17)
Peripheral Vascular	33.54	-13.73	6.31	26.13	71.56	97.69
	(0.59)	(0.34)	(0.75)	(0.73)	(0.58)	(0.55)
Cerebrovascular.	133.40	-19.22	66.17	180.36	449.48**	629.84***
	(0.30)	(0.32)	(0.30)	(0.16)	(0.01)	(0.00)
Chronic Pulmonary	-50.14	28.26***	-16.20	-38.08	22.74	-15.34
	(0.26)	(0.00)	(0.34)	(0.45)	(0.80)	(0.89)
Peptic Ulcer	-115.46	6.40	-28.30	-137.36*	-23.62	-160.97
	(0.07)	(0.61)	(0.14)	(0.04)	(0.84)	(0.22)
Mild Liver	327.60	46.20	-24.38	349.43	732.60	1082.03
	(0.09)	(0.06)	(0.40)	(0.13)	(0.19)	(0.06)
Diabetes w/o Complications	59.26	-12.63	-6.47	40.15	242.44	282.60
	(0.43)	(0.39)	(0.29)	(0.61)	(0.08)	(0.07)
Diabetes w. Complications	-54.46	24.15	-22.54	-52.86	56.76	3.91
	(0.31)	(0.20)	(0.39)	(0.50)	(0.64)	(0.98)
Renal Disease	46.34	-26.85	102.20	121.69	405.86	527.55
	(0.74)	(0.31)	(0.37)	(0.62)	(0.08)	(0.12)
Cancer	117.06	-29.46	-29.72	57.89	22.94	80.83
	(0.25)	(0.50)	(0.09)	(0.46)	(0.87)	(0.60)
Moderate/Severe Liver	-135.79	-95.19	9.99	-220.98	-494.69	-715.68
	(0.54)	(0.07)	(0.85)	(0.37)	(0.52)	(0.36)
Metastatic. Carcinoma	205.19	27.96	5.65	238.80	377.87	616.66*
	(0.27)	(0.55)	(0.84)	(0.18)	(0.13)	(0.02)

Cardiac Arrhythmia	27.22	58.98*	17.40	103.59	42.30	145.89
	(0.80)	(0.05)	(0.58)	(0.46)	(0.85)	(0.51)
Hypertension (Uncomplicated)	84.43	-8.52	-21.27	54.64	-12.13	42.51
	(0.16)	(0.58)	(0.07)	(0.35)	(0.86)	(0.65)
Hypothyroidism	67.60	17.31	-13.26	71.65	322.46	394.11
	(0.50)	(0.47)	(0.13)	(0.55)	(0.16)	(0.07)
Depression/Psychoses	-15.41	17.27	-1.77	0.09	50.54	50.63
	(0.76)	(0.08)	(0.91)	(1.00)	(0.47)	(0.59)
Obesity	-138.57	19.78	-65.37	-184.16	-506.57*	-690.72**
	(0.27)	(0.56)	(0.24)	(0.28)	(0.04)	(0.01)
Constant	-3.05	-16.10***	4.71	-14.45	-1.78	-16.23
	(0.83)	(0.00)	(0.28)	(0.35)	(0.96)	(0.69)

Note: p-values are presented in parenthesis.* p-value≤0.05, ** p-value≤0.01, *** p-value≤0.001

Appendix Table D.2. Regression results for cost outcomes, 2006 vs. 2009

	Chronic Man. Drugs	Acute Drugs	Asthma Drugs (Unc.)	Asthma & COPD Drugs	Other Drugs	Total Drugs
Intervention effect	178.55***	-10.35	24.05	192.24***	51.39	243.64*
	(0.00)	(0.18)	(0.18)	(0.00)	(0.53)	(0.02)
Tel. Contact w. MD	21.74	6.10	-1.90	25.94	-59.09	-33.15
	(0.32)	(0.26)	(0.63)	(0.27)	(0.55)	(0.75)
Non-Clinical MD Serv.	5.56	-3.16	-11.20	-8.79	-38.33	-47.12
	(0.86)	(0.76)	(0.09)	(0.81)	(0.62)	(0.62)
Imaging Serv.	59.70*	5.31	6.61	71.62**	-55.42	16.21
	(0.02)	(0.16)	(0.40)	(0.01)	(0.06)	(0.63)
Other Slush Code Serv.	0.73	0.06	-0.18	0.62	10.10	10.71
	(0.79)	(0.88)	(0.71)	(0.84)	(0.22)	(0.26)
Myocardial Infarction	204.03	44.05	-22.57	225.51	567.71*	793.21
	(0.22)	(0.37)	(0.38)	(0.31)	(0.04)	(0.06)
Cong. Heart Failure	84.10	1.71	1.26	87.07	266.97	354.04*
	(0.35)	(0.96)	(0.93)	(0.35)	(0.10)	(0.05)
Peripheral Vascular	123.47	-39.96	-1.00	82.51	213.69	296.20
	(0.36)	(0.08)	(0.96)	(0.58)	(0.30)	(0.27)
Cerebrovascular	-11.87	-43.84**	71.96	16.25	546.71***	562.96***
	(0.90)	(0.01)	(0.26)	(0.86)	(0.00)	(0.00)
Chronic Pulmonary	-104.22*	30.41**	-13.19	-87.00	36.93	-50.07
	(0.05)	(0.01)	(0.41)	(0.17)	(0.71)	(0.69)
Peptic Ulcer	-89.59	-10.75	-18.28	-118.62	15.96	-102.65
	(0.17)	(0.47)	(0.18)	(0.09)	(0.90)	(0.45)
Mild Liver	301.23	32.68	-12.32	321.59	896.86	1218.45*
	(0.17)	(0.15)	(0.61)	(0.18)	(0.12)	(0.05)
Diabetes w/o Complications	38.11	-2.93	2.32	37.50	270.24	307.73
	(0.62)	(0.88)	(0.76)	(0.68)	(0.11)	(0.12)
Diabetes w. Complications	-51.68	21.87	-13.66	-43.47	-174.37	-217.84
	(0.50)	(0.29)	(0.57)	(0.66)	(0.19)	(0.22)
Renal Disease	-58.04	14.84	60.92	17.73	360.17	377.89
	(0.65)	(0.62)	(0.44)	(0.93)	(0.18)	(0.24)
Cancer	125.87	-19.10	-17.61	89.17	144.76	233.93
	(0.29)	(0.63)	(0.19)	(0.36)	(0.26)	(0.12)
Moderate/Severe Liver	-352.95	-81.96	13.69	-421.23	8.67	-412.56
	(0.17)	(0.17)	(0.75)	(0.18)	(0.99)	(0.71)
Metastatic. Carcinoma	421.87	43.53	-4.75	460.64	800.49***	1261.1***
	(0.07)	(0.34)	(0.83)	(0.06)	(0.00)	(0.00)
Cardiac Arrhythmia	91.13	47.75	14.00	152.87	162.74	315.61
	(0.50)	(0.16)	(0.67)	(0.39)	(0.55)	(0.32)

Hypertension (Uncomplicated)	84.71	4.34	-17.59	71.46	120.37	191.83
	(0.20)	(0.75)	(0.10)	(0.29)	(0.17)	(0.08)
Hypothyroidism	10.95	4.07	-11.59	3.42	86.17	89.59
	(0.92)	(0.85)	(0.15)	(0.98)	(0.56)	(0.64)
Depression/Psychoses	8.01	11.68	12.99	32.68	158.99	191.68
	(0.89)	(0.31)	(0.47)	(0.67)	(0.06)	(0.11)
Obesity	-117.22	52.29	-50.62	-115.55	-611.97	-727.52
	(0.36)	(0.53)	(0.25)	(0.54)	(0.07)	(0.10)
Constant	3.78	-14.55***	0.42	-10.34	-56.28	-66.62
	(0.82)	(0.00)	(0.92)	(0.55)	(0.29)	(0.24)

Note: p-values are presented in parenthesis.* p-value≤0.05, ** p-value≤0.01, *** p-value≤0.001

Appendix Table D.3 Regression results for cost outcomes, 2006 vs. 2010

	Chronic Man. Drugs	Acute Drugs	Asthma Drugs (Unc.)	Asthma & COPD Drugs	Other Drugs	Total Drugs
Intervention effect	195.53***	-6.09	15.50	204.94***	168.21	373.15**
	(0.00)	(0.48)	(0.35)	(0. 00)	(0.15)	(0.01)
Tel. Contact w. MD	-144.19*	0.37	-2.77	-146.58*	-164.70	-311.28
	(0.03)	(0.96)	(0.42)	(0.04)	(0.64)	(0.37)
Non-Clinical MD Serv.	0.14	-7.34	-14.0**	-21.25	-45.69	-66.94
	(1.00)	(0.41)	(0.01)	(0.70)	(0.77)	(0.71)
Imaging Serv.	75.83	12.73***	-1.38	87.18	-41.88	45.30
	(0.13)	(0.00)	(0.79)	(0.08)	(0.60)	(0.45)
Other Slush Code Serv.	6.03	0.80	0.71	7.54	-21.48	-13.94
	(0.23)	(0.48)	(0.48)	(0.08)	(0.14)	(0.35)
Myocardial Infarction	41.90	111.18	-13.83	139.26	2350.42	2489.68
	(0.71)	(0.10)	(0.43)	(0.26)	(0.17)	(0.16)
Cong. Heart Failure	256.12	-15.84	5.29	245.57	201.45	447.02
	(0.15)	(0.64)	(0.66)	(0.13)	(0.41)	(0.12)
Peripheral Vascular	83.66	-6.29	-5.30	72.08	349.24	421.32
	(0.23)	(0.81)	(0.77)	(0.41)	(0.20)	(0.11)
Cerebrovascular	-23.58	-36.19	72.15	12.38	549.43*	561.81*
	(0.87)	(0.10)	(0.26)	(0.91)	(0.05)	(0.05)
Chronic Pulmonary	-177.4***	25.49	-16.07	-168.0***	512.63	344.62
	(0.00)	(0.08)	(0.31)	(0.00)	(0.14)	(0.35)
Peptic Ulcer	-94.48	-19.22	1.58	-112.12	69.96	-42.16
	(0.26)	(0.34)	(0.90v	(0.22)	(0.74)	(0.85)
Mild Liver	353.90*	55.88	-20.53	389.24*	1378.49	1767.74
	(0.03)	(0.08)	(0.42)	(0.05)	(0.15)	(0.06)
Diabetes w/o Complications	-4.66	10.03	-3.67	1.70	1213.16	1214.86
	(0.94)	(0.72)	(0.60)	(0.98)	(0.08)	(0.09)
Diabetes w. Complications	-71.60	6.53	-8.07	-73.14	-177.96	-251.10
	(0.42)	(0.76)	(0.69)	(0.43)	(0.39)	(0.27)
Renal Disease	-187.00	-22.07	-17.04	-226.11	126.11	-100.00
	(0.12)	(0.55)	(0.38)	(0.07)	(0.75)	(0.79)
Cancer	101.85	-17.11	0.74	85.48	-35.13	50.34
	(0.40)	(0.64)	(0.95)	(0.42)	(0.90)	(0.86)
Moderate/Severe Liver	-465.36*	-111.23	39.28	-537.32*	-652.32	-1189.64
	(0.04)	(0.19)	(0.28)	(0.04)	(0.58)	(0.33)
Metastatic. Carcinoma	550.06*	110.92	-17.37	643.60*	720.91	1364.51
	(0.03)	(0.20)	(0.41)	(0.04)	(0.25)	(0.10)
Cardiac Arrhythmia	229.76	82.95	11.17	323.88	-0.07	323.81
	(0.20)	(0.17)	(0.72)	(0.09)	(1.00)	(0.48)

Hypertension (Uncomplicated)	72.97	1.64	-10.07	64.55	45.22	109.77
	(0.25)	(0.93)	(0.31)	(0.31)	(0.81)	(0.57)
Hypothyroidism	13.91	-3.13	-6.25	4.52	-174.88	-170.36
	(0.89)	(0.86)	(0.46)	(0.97)	(0.62)	(0.69)
Depression/Psychoses	-40.46	1.17	27.01	-12.28	44.17	31.89
	(0.45)	(0.91)	(0.25)	(0.83)	(0.82)	(0.88)
Obesity	-144.64	44.20	57.99	-42.45	-123.17	-165.62
	(0.28)	(0.61)	(0.17)	(0.83)	(0.77)	(0.72)
Constant	27.38	-11.86**	-0.92	14.60	-122.32	-107.72
	(0.10)	(0.01)	(0.81)	(0.39)	(0.11)	(0.17)

Note: p-values are presented in parenthesis.* p-value≤0.05, ** p-value≤0.01, *** p-value≤0.001

Appendix Table D.4 Regression results for cost outcomes, 2006 vs. 2011

	Chronic Man. Drugs	Acute Drugs	Asthma Drugs (Unc.)	Asthma & COPD Drugs	Other Drugs	Total Drugs
Intervention effect	157.09***	-8.50	8.03	156.62***	109.13	265.75
	(0.00)	(0.40)	(0.60)	(0.00)	(0.38)	(0.06)
Tel. Contact w. MD	-70.70	-2.05	-1.40	-74.16*	50.30	-23.86
	(0.03)	(0.69)	(0.60)	(0.03)	(0.39)	(0.67)
Non-Clinical MD Serv.	-30.13	-3.19	-8.10	-41.42	-13.57	-55.00
	(0.31)	(0.72)	(0.08)	(0.27)	(0.86)	(0.51)
Imaging Serv.	58.96**	8.76	3.22	70.94**	43.56	114.51
	(0.01)	(0.29)	(0.51)	(0.01)	(0.81)	(0.53)
Other Slush Code Serv.	3.55	1.10	0.92	5.57	22.64	28.21
	(0.42)	(0.29)	(0.33)	(0.22)	(0.32)	(0.23)
Myocardial Infarction	-93.69	28.61	-17.63	-82.71	2068.54	1985.83
	(0.30)	(0.62)	(0.18)	(0.45)	(0.14)	(0.16)
Cong. Heart Failure	248.06	-21.31	5.43	232.18	342.10	574.28
	(0.11)	(0.59)	(0.66)	(0.12)	(0.23)	(0.11)
Peripheral Vascular	-21.67	-5.87	-1.14	-28.68	325.11	296.43
	(0.81)	(0.83)	(0.94)	(0.78)	(0.23)	(0.27)
Cerebrovascular	-22.33	-30.97	55.61	2.31	478.02	480.32
	(0.86)	(0.26)	(0.29)	(0.98)	(0.09)	(0.09)
Chronic Pulmonary	-142.7***	36.42**	-12.35	-118.59*	492.51	373.92
	(0.00)	(0.01)	(0.34)	(0.03)	(0.08)	(0.21)
Peptic Ulcer	-69.00	10.74	-10.27	-68.53	291.88	223.35
	(0.38)	(0.60)	(0.23)	(0.38)	(0.27)	(0.40)
Mild Liver	355.11	173.18	-19.44	508.85	1395.23	1904.09
	(0.09)	(0.25)	(0.31)	(0.06)	(0.19)	(0.09)
Diabetes w/o Complications	-31.71	7.93	-4.21	-27.99	1081.98	1053.99
	(0.66)	(0.73)	(0.32)	(0.69)	(0.07)	(0.08)
Diabetes w. Complications	70.85	27.21	7.59	105.65	-276.89	-171.24
	(0.53)	(0.32)	(0.69)	(0.36)	(0.23)	(0.51)
Renal Disease	-299.54**	-56.53	-29.22	-385.29**	132.10	-253.19
	(0.01)	(0.12)	(0.06)	(0.01)	(0.80)	(0.62)
Cancer	131.85	-5.37	6.48	132.96	-295.99	-163.03
	(0.28)	(0.87)	(0.61)	(0.22)	(0.25)	(0.56)
Moderate/Severe Liver	-762.7***	-202.13	34.66	-930.2***	-1557.27	-2487.48
	(0.00)	(0.21)	(0.34)	(0.00)	(0.41)	(0.19)
Metastatic. Carcinoma	589.86*	58.82	-21.55	627.13*	561.59	1188.72
	(0.03)	(0.41)	(0.22)	(0.04)	(0.29)	(0.10)
Cardiac Arrhythmia	312.80	154.32	18.50	485.62*	55.96	541.58
	(0.07)	(0.10)	(0.53)	(0.03)	(0.91)	(0.28)

Hypertension (Uncomplicated)	146.46*	31.06	-4.19	173.33*	171.13	344.46*
	(0.03)	(0.29)	(0.64)	(0.02)	(0.25)	(0.04)
Hypothyroidism	50.29	-13.50	-3.47	33.32	25.52	58.84
	(0.61)	(0.40)	(0.59)	(0.76)	(0.95)	(0.90)
Depression/Psychoses	-23.82	10.01	8.85	-4.96	70.65	65.69
	(0.68)	(0.46)	(0.51)	(0.94)	(0.69)	(0.75)
Obesity	-113.69	20.10	-21.81	-115.40	-136.95	-252.35
	(0.47)	(0.78)	(0.09)	(0.57)	(0.79)	(0.64)
Constant	26.22	-16.90***	0.39	9.71	-107.25	-97.55
	(0.11)	(0.00)	(0.92)	(0.60)	(0.14)	(0.19)

Note: p-values are presented in parenthesis.* p-value≤0.05, ** p-value≤0.01, *** p-value≤0.001

Appendix Table G.1 Regression results for drug dispensation outcomes, 2006 vs. 2008

	Chronic management	Acute	Asthma	Asthma\COPD	Other	Total
Intervention effect	1.52***	0.03	0.58*	2.12***	-0.73	1.39
	(0.00)	(0.91)	(0.02)	(0.00)	(0.54)	(0.34)
Tel. Contact w. MD	1.39	0.17	0.04	1.60	4.90***	6.50***
	(0.12)	(0.43)	(0.74)	(0.10)	(0.00)	(0.00)
Non-Clinical MD Serv.	-0.73	0.15	-0.39	-0.96	1.31	0.34
	(0.09)	(0.37)	(0.07)	(0.11)	(0.48)	(0.87)
Imaging Serv.	0.90**	0.63***	0.15	1.69***	0.36	2.04**
	(0.01)	(0.00)	(0.15)	(0.00)	(0.46)	(0.01)
Other Slush Code Serv.	-0.03	-0.02	0.00	-0.05	0.27*	0.22
	(0.46)	(0.54)	(0.91)	(0.48)	(0.04)	(0.18)
Myocardial Infarction	0.70	2.46	-0.66	2.50	(10.17**	12.67*
	(0.73)	(0.13)	(0.09)	(0.49)	0.01)	(0.04)
Cong. Heart Failure	1.01	0.77	0.03	1.81	8.91***	10.72***
	(0.22)	(0.21)	(0.90)	(0.15)	(0.00)	(0.00)
Peripheral Vascular	0.43	0.13	0.22	0.77	-0.03	0.74
	(0.65)	(0.76)	(0.48)	(0.52)	(0.99)	(0.78)
Cerebrovascular	0.73	-1.42*	1.17	0.48	9.38***	9.86***
	(0.61)	(0.03)	(0.14)	(0.77)	(0.00)	(0.00)
Chronic Pulmonary	-0.22	0.98*	-0.30	0.46	0.91	1.38
	(0.68)	(0.02)	(0.19)	(0.58)	(0.64)	(0.56)
Peptic Ulcer	-0.87	0.33	-0.43	-0.97	-2.53	-3.49
	(0.29)	(0.49)	(0.12)	(0.30)	(0.41)	(0.30)
Mild Liver	3.24	0.93	0.07	4.24	3.07	7.31**
	(0.11)	(0.19)	(0.94)	(0.15)	(0.19)	(0.01)
Diabetes w/o Complications	0.39	0.14	-0.11	0.42	1.87	2.29
	(0.66)	(0.85)	(0.30)	(0.76)	(0.32)	(0.38)
Diabetes w. Complications	-0.61	0.27	-0.54	-0.87	1.65	0.77
	(0.53)	(0.68)	(0.15)	(0.56)	(0.55)	(0.77)
Renal Disease	-0.04	-1.47	1.57	0.07	8.02**	8.09
	(0.98)	(0.13)	(0.36)	(0.98)	(0.01)	(0.11)
Cancer	1.10	-0.84	-0.46	-0.20	-0.90	-1.11
	(0.20)	(0.54)	(0.07)	(0.88)	(0.68)	(0.68)
Moderate/Severe Liver	-0.38	-5.14	-0.15	-5.67	13.39	7.72
	(0.89)	(0.15)	(0.89)	(0.16)	(0.54)	(0.75)
Metastatic. Carcinoma	3.69	1.25	0.20	5.14*	6.41	11.55*
	(0.10)	(0.43)	(0.64)	(0.05)	(0.15)	(0.02)

Table G1. Continued

Cardiac Arrhythmia	0.44	2.70***	0.17	3.31	-1.28	2.03
	(0.78)	(0.00)	(0.64)	(0.10)	(0.62)	(0.49)
Hypertension (Uncomplicated)	1.04	-0.20	-0.33*	0.51	0.78	1.29
	(0.10)	(0.65)	(0.05)	(0.50)	(0.57)	(0.42)
Hypothyroidism	0.45	-0.22	-0.20	0.02	6.17	6.19
	(0.67)	(0.61)	(0.12)	(0.99)	(0.16)	(0.18)
Depression/Psychoses	-0.10	0.43	0.12	0.45	1.69	2.14
	(0.86)	(0.17)	(0.69)	(0.60)	(0.26)	(0.23)
Obesity	-1.63	1.40	-1.13	-1.37	-6.60	-7.97
	(0.30)	(0.16)	(0.22)	(0.55)	(0.12)	(0.12)
Constant	0.05	-0.61***	0.03	-0.54**	0.58	0.05
	(0.76)	(0.00)	(0.63)	(0.01)	(0.25)	(0.93)

Note: p-values are presented in parenthesis.* p-value≤0.05, ** p-value≤0.01, *** p-value≤0.001

Appendix Table G.2 Regression results for drug dispensation outcomes, 2006 vs. 2009

	Chronic management	Acute	Asthma	Asthma\COPD	Other	Total
Intervention effect	1.66***	-0.12	0.41	1.95***	0.78	2.73
	(0.00)	(0.69)	(0.06)	(0.00)	(0.55)	(0.09)
Tel. Contact w. MD	0.25	0.35	0.02	0.61	0.76	1.37
	(0.37)	(0.11)	(0.78)	(0.08)	(0.61)	(0.33)
Non-Clinical MD Serv.	-0.10	0.43	-0.36*	-0.04	-0.33	-0.37
	(0.83)	(0.28)	(0.04)	(0.96)	(0.87)	(0.87)
Imaging Serv.	0.75**	0.24	0.21	1.20***	-0.35	0.85
	(0.01)	(0.11)	(0.07)	(0.00)	(0.56)	(0.18)
Other Slush Code Serv.	0.01	0.01	-0.01	0.01	0.35**	0.36*
	(0.77)	(0.58)	(0.31)	(0.82)	(0.01)	(0.02)
Myocardial Infarction	1.48	1.22	-0.16	2.54	14.44***	16.97**
	(0.55)	(0.39)	(0.77)	(0.54)	(0.00)	(0.01)
Cong. Heart Failure	1.13	0.13	0.01	1.26	9.66***	10.91***
	(0.30)	(0.84)	(1.00)	(0.36)	(0.00)	(0.00)
Peripheral Vascular	1.60	-0.64	0.05	1.01	3.45	4.46
	(0.35)	(0.32)	(0.85)	(0.62)	(0.39)	(0.38)
Cerebrovascular	-0.58	-2.27***	1.35	-1.50	10.94***	9.44***
	(0.60)	(0.00)	(0.11)	(0.31)	(0.00)	(0.00)
Chronic Pulmonary	-0.98	0.86	-0.08	-0.20	-0.72	-0.92
	(0.10)	(0.06)	(0.75)	(0.84)	(0.73)	(0.73)
Peptic Ulcer	-0.81	-0.01	-0.26	-1.08	-1.75	-2.83
	(0.32)	(0.99)	(0.26)	(0.32)	(0.61)	(0.42)
Mild Liver	3.73	0.95	0.36	5.03	6.38*	11.41*
	(0.12)	(0.45)	(0.64)	(0.13)	(0.05)	(0.03)
Diabetes w/o Complications	0.14	0.19	0.19	0.52	2.23	2.76
	(0.88)	(0.79)	(0.32)	(0.74)	(0.28)	(0.35)
Diabetes w. Complications	-0.96	0.29	-0.36	-1.04	-2.78	-3.82
	(0.40)	(0.61)	(0.28)	(0.50)	(0.35)	(0.23)
Renal Disease	-1.07	0.26	0.91	0.10	6.45*	6.56
	(0.53)	(0.79)	(0.45)	(0.97)	(0.03)	(0.08)
Cancer	1.53	-0.58	-0.27	0.68	2.40	3.08
	(0.21)	(0.59)	(0.18)	(0.56)	(0.22)	(0.15)
Moderate/Severe Liver	-2.98	-3.94	-0.16	-7.08	11.19	4.12
	(0.27)	(0.18)	(0.87)	(0.17)	(0.64)	(0.88)
Metastatic. Carcinoma	6.35*	2.71	-0.08	8.98**	20.28***	29.26***
	(0.02)	(0.11)	(0.83)	(0.01)	(0.00)	(0.00)
Cardiac Arrhythmia	1.23	1.52	0.07	2.82	1.73	4.54
	(0.53)	(0.12)	(0.86)	(0.28)	(0.61)	(0.25)

Table G2. Continued

Hypertension (Uncomplicated)	1.05	0.44	-0.27	1.22	4.21**	5.43**
	(0.16)	(0.44)	(0.07)	(0.23)	(0.01)	(0.01)
Hypothyroidism	-0.38	-0.21	-0.16	-0.76	9.46	8.70
	(0.74)	(0.82)	(0.18)	(0.69)	(0.07)	(0.10)
Depression/Psychoses	0.18	0.33	0.29	0.80	3.93	4.73*
	(0.79)	(0.40)	(0.40)	(0.46)	(0.06)	(0.05)
Obesity	-1.52	2.03	-1.06	-0.55	-11.92	-12.46
	(0.44)	(0.39)	(0.19)	(0.88)	(0.14)	(0.22)
Constant	0.14	-0.50***	-0.06	-0.43	0.26	-0.16
	(0.44)	(0.00)	(0.34)	(0.08)	(0.65)	(0.80)

Note: p-values are presented in parenthesis.* p-value≤0.05, ** p-value≤0.01, *** p-value≤0.001

Appendix Table G.3 Regression results for drug dispensation outcomes, 2006 vs. 2010

	Chronic management	**Acute**	**Asthma**	**Asthma\COPD**	**Other**	**Total**
Intervention effect	1.62***	0.11	0.27	2.01***	0.49	2.50
	(0.00)	(0.74)	(0.18)	(0.00)	(0.71)	(0.14)
Tel. Contact w. MD	-0.91*	-0.13	-0.04	-1.08	3.23*	2.15
	(0.04)	(0.74)	(0.36)	(0.16)	(0.02)	(0.28)
Non-Clinical MD Serv.	-0.44	0.05	-0.42**	-0.82	-2.68	-3.50
	(0.52)	(0.88)	(0.01)	(0.29)	(0.23)	(0.19)
Imaging Serv.	0.70	0.81***	0.00	1.50**	1.60	3.10*
	(0.14)	(0.00)	(0.95)	(0.01)	(0.07)	(0.02)
Other Slush Code Serv.	0.06	0.03	0.00	0.10	0.08	0.17
	(0.18)	(0.54)	(0.72)	(0.07)	(0.65)	(0.33)
Myocardial Infarction	-0.33	2.71	-0.04	2.34	21.47***	23.81***
	(0.79)	(0.11)	(0.90)	(0.31)	(0.00)	(0.00)
Cong. Heart Failure	2.21	-0.08	0.08	2.21	8.80**	11.01***
	(0.18)	(0.93)	(0.60)	(0.22)	(0.01)	(0.00)
Peripheral Vascular.	0.74	-0.33	0.03	0.43	7.29	7.72
	(0.41)	(0.58)	(0.91)	(0.74)	(0.15)	(0.13)
Cerebrovascular	-0.80	-2.35**	1.15	-2.00	13.46***	11.46***
	(0.56)	(0.01)	(0.14)	(0.16)	(0.00)	(0.00)
Chronic Pulmonary	-1.49***	0.41	-0.14	-1.22	1.58	0.36
	(0.00)	(0.42)	(0.56)	(0.16)	(0.50)	(0.90)
Peptic Ulcer	-0.78	-0.18	0.02	-0.95	-0.01	-0.96
	(0.35)	(0.83)	(0.93)	(0.45)	(1.00)	(0.81)
Mild Liver	4.33**	1.12	-0.18	5.28	6.12*	11.40***
	(0.01)	(0.49)	(0.79)	(0.12)	(0.02)	(0.00)
Diabetes w/o Complications	-0.49	0.03	0.07	-0.39	5.61*	5.22
	(0.43)	(0.97)	(0.55)	(0.72)	(0.02)	(0.08)
Diabetes w. Complications	-0.86	0.46	-0.16	-0.57	-2.65	-3.22
	(0.45)	(0.57)	(0.54)	(0.69)	(0.40)	(0.33)
Renal Disease	-2.04	-1.79	-0.48	-4.31*	3.28	-1.03
	(0.17)	(0.17)	(0.15)	(0.02)	(0.33)	(0.80)
Cancer	1.25	-0.14	-0.07	1.04	4.30	5.34
	(0.20)	(0.90)	(0.59)	(0.51)	(0.11)	(0.09)
Moderate/Severe Liver	-3.82	-4.41	0.56	-7.68	7.69	0.02
	(0.10)	(0.20)	(0.42)	(0.08)	(0.69)	(1.00)
Metastatic. Carcinoma	8.25***	5.65	-0.24	13.67*	22.94***	36.61***
	(0.00)	(0.11)	(0.39)	(0.02)	(0.00)	(0.00)
Cardiac Arrhythmia	2.26	2.39	0.09	4.73	-1.25	3.48
	(0.27)	(0.23)	(0.81)	(0.12)	(0.75)	(0.48)

Table G3. Continued

Hypertension (Uncomplicated)	0.61	0.71	-0.11	1.22	5.29***	6.51***
	(0.28)	(0.22)	(0.42)	(0.19)	(0.00)	(0.00)
Hypothyroidism	-0.07	-0.07	-0.09	-0.23	6.94	6.71
	(0.95)	(0.93)	(0.39)	(0.89)	(0.06)	(0.13)
Depression/Psychoses	-0.28	0.16	0.42	0.30	3.82	4.12
	(0.57)	(0.65)	(0.25)	(0.69)	(0.07)	(0.07)
Obesity	-2.11	1.01	0.43	-0.67	-4.14	-4.82
	(0.27)	(0.58)	(0.27)	(0.84)	(0.57)	(0.49)
Constant	0.29	-0.37*	-0.08	-0.16	0.79	0.63
	(0.07)	(0.04)	(0.13)	(0.53)	(0.21)	(0.37)

Note: p-values are presented in parenthesis.* p-value≤0.05, ** p-value≤0.01, *** p-value≤0.001

Appendix Table G.4 Regression results for drug dispensation outcomes, 2006 vs. 2011

	Chronic management	Acute	Asthma	Asthma\COPD	Other	Total
Intervention effect	1.45***	-0.17	0.17	1.45*	1.79	3.23
	(0.00)	(0.60)	(0.37)	(0.03)	(0.24)	(0.09)
Tel. Contact w. MD	-0.68*	0.10	0.01	-0.57	1.62	1.05
	(0.05)	(0.49)	(0.98)	(0.14)	(0.13)	(0.27)
Non-Clinical MD Serv.	-0.62	0.21	-0.19	-0.60	-0.75	-1.35
	(0.24)	(0.29)	(0.04)	(0.33)	(0.45)	(0.31)
Imaging Serv.	0.58*	0.42	0.07	1.07**	0.25	1.32
	(0.02)	(0.09)	(0.28)	(0.01)	(0.74)	(0.20)
Other Slush Code Serv.	0.02	0.05	0.01	0.08	0.10	0.18
	(0.73)	(0.18)	(0.40)	(0.26)	(0.46)	(0.30)
Myocardial Infarction	-1.29	1.57	-0.25	0.03	21.70***	21.74**
	(0.43)	(0.35)	(0.16)	(0.99)	(0.00)	(0.01)
Cong. Heart Failure	2.25	-0.75	0.08	1.57	14.83**	16.40*
	(0.16)	(0.43)	(0.64)	(0.40)	(0.01)	(0.02)
Peripheral Vascular	-0.44	-0.01	0.08	-0.37	3.67	3.30
	(0.68)	(0.99)	(0.71)	(0.80)	(0.50)	(0.57)
Cerebrovascular	-0.75	-2.18*	0.73	-2.20	10.35***	8.15*
	(0.59)	(0.03)	(0.24)	(0.17)	(0.00)	(0.02)
Chronic Pulmonary	-0.98	1.18*	-0.20	0.01	1.48	1.48
	(0.07)	(0.02)	(0.26)	(1.00)	(0.56)	(0.64)
Peptic Ulcer	-0.28	0.97	-0.11	0.58	4.92	5.50
	(0.78)	(0.21)	(0.44)	(0.64)	(0.15)	(0.11)
Mild Liver	4.58	0.70	-0.14	5.14	1.28	6.42
	(0.06)	(0.72)	(0.77)	(0.15)	(0.66)	(0.19)
Diabetes w/o Complications	-0.97	0.34	-0.03	-0.66	8.20***	7.54*
	(0.21)	(0.70)	(0.58)	(0.56)	(0.00)	(0.02)
Diabetes w. Complications	0.63	1.20	0.09	1.92	0.95	2.87
	(0.66)	(0.14)	(0.72)	(0.27)	(0.77)	(0.49)
Renal Disease	-3.46*	-2.31	-0.60*	-6.38***	-1.77	-8.14
	(0.03)	(0.07)	(0.05)	(0.00)	(0.63)	(0.08)
Cancer	1.26	0.90	0.05	2.21	1.40	3.61
	(0.19)	(0.24)	(0.71)	(0.11)	(0.64)	(0.32)
Moderate/Severe Liver	-7.44**	-4.11	0.50	-11.05**	21.79	10.74
	(0.01)	(0.20)	(0.44)	(0.01)	(0.21)	(0.58)
Metastatic. Carcinoma	8.88***	2.98	-0.30	11.56*	13.77*	25.33*
	(0.00)	(0.26)	(0.21)	(0.03)	(0.04)	(0.02)
Cardiac Arrhythmia	3.28	3.43	0.26	6.97	-0.16	6.81
	(0.10)	(0.15)	(0.50)	(0.06)	(0.97)	(0.21)

Table G4. Continued

Hypertension (Uncomplicated)	1.62*	0.87	-0.04	2.44*	6.83***	9.28***
	(0.02)	(0.15)	(0.71)	(0.02)	(0.00)	(0.00)
Hypothyroidism	0.21	-0.52	-0.02	-0.33	10.22*	9.88
	(0.84)	(0.51)	(0.84)	(0.84)	(0.02)	(0.07)
Depression/Psychoses	-0.12	0.54	0.27	0.69	4.98	5.66
	(0.84)	(0.14)	(0.34)	(0.45)	(0.06)	(0.08)
Obesity	-1.69	-0.02	-0.63	-2.34	-3.89	-6.23
	(0.44)	(0.99)	(0.06)	(0.44)	(0.54)	(0.42)
Constant	0.09	-0.66***	-0.05	-0.62*	1.22	0.60
	(0.60)	(0.00)	(0.26)	(0.02)	(0.07)	(0.45)

Note: p-values are presented in parenthesis.* p-value≤0.05, ** p-value≤0.01, *** p-value≤0.001
